# Effect of aromatherapy with peppermint essential oil on the gag reflex: a randomized, placebo-controlled, single-blind, crossover study

**DOI:** 10.1186/s12906-024-04334-3

**Published:** 2024-01-27

**Authors:** Ayuko Okamoto, Hiroyuki Karibe, Satoshi Tanaka, Yuichi Kato, Tomomi Kawakami, Yutaka Okamoto, Greg Goddard

**Affiliations:** 1 Department of Pediatric Dentistry, The Nippon Dental University School of Life Dentistry at Tokyo, 1- 9-20 Fujimi, Chiyoda-ku, Tokyo, 102-8159 Japan; 2 Department of Dental Anesthesiology, The Nippon Dental University School of Life Dentistry at Tokyo, 1-9-20 Fujimi, Chiyoda-ku, Tokyo, 102-8159 Japan; 3 University of California, San Francisco, 707 Parnassus Avenue, San Francisco, CA 94143 USA

**Keywords:** Dental anxiety, Gagging, Nitrous oxide, Pharyngeal reflex, Vomiting

## Abstract

**Background:**

Sensitive gag reflexes prevent dental patients from receiving appropriate treatment. Aromatherapy helps patients relax during dental procedures. However, the effect of aromatherapy on the gag reflex caused by the stimulation of the oral cavity is unknown. This study aimed to evaluate whether aromatherapy reduces gag reflexes during oral stimulation.

**Methods:**

In this randomized, placebo-controlled, single-blind, crossover study, the gag reflexes of 24 healthy individuals (12 females and 12 males; mean age: 34.3 ± 9.5 years) were quantified. A standard saliva ejector was slowly guided down the participant’s throat to determine the maximum tolerance of the gag reflex, and the insertion distance was measured to quantify the gag reflex. All individuals participated in an aromatherapy session with peppermint essential oil and a placebo session with distilled water. The gag reflex was quantified before (baseline) and after each session. Another measurement was performed using nitrous oxide/oxygen inhalation as a positive control.

**Results:**

Gag reflex values significantly increased after aromatherapy with both peppermint essential oil and placebo compared to baseline values (paired *t*-test, *P* < 0.001 and *P* = 0.014, respectively). The gag reflex value also increased significantly during nitrous oxide/oxygen inhalation (paired *t*-test, *P* < 0.001). There was no significant difference in the increase rate of gag reflex values between the positive control and aromatherapy interventions, but it was significantly lower after the placebo intervention (repeated measures analysis of variance, *P* = 0.003; post-hoc test, *P* = 0.83 and *P* = 0.02).

**Conclusion:**

Aromatherapy with peppermint essential oil has the potential for reducing gag reflex during dental procedures.

**Trial registration:**

The study was registered in the University hospital Medical Information Network Clinical Trials Registry under the code UMIN000050616 (approved 17/03/2023).

## Background

In dental practice, many patients experience discomfort such as gag reflex [1]. The gag reflex caused by the stimulation of the oral cavity prevents safe and smooth dental treatment [2]. According to a recent survey, 8.2% of patients complained of choking and nausea during dental treatment [3]. Gagging in the dental office is strongly related to anxiety about dental treatment [4]. If emesis during dental treatment becomes traumatic, patients tend to avoid dental visits, resulting in maladaptive behaviors during dental treatment. Consequently, patients fail to receive early treatment for dental diseases. Long-term avoidance of treatment significantly lowers the quality of life. Therefore, to improve the quality of dental care, it is crucial to investigate non-invasive and reliable strategies for controlling the gag reflex in patients.

Drug therapy, especially anti-anxiety drugs, and sedation via inhalation of nitrous oxide and intravenous sedatives have been reported to provide relief from the gag reflex [5–7]. However, it is necessary to consider the side effects of drugs and the recovery time after sedation. In pediatric dentistry, the gag reflex of children is minimized by allowing them to watch videos, listen to music, and directing their attention to external elements [8]. Recently, acupressure at acupuncture points and percutaneous electrical stimulation have been used as relaxation methods in clinical dental practice [9, 10]. However, few studies have provided scientific evidence for the effects of these strategies on the gag reflex [5]. An appropriate evaluation of the gag reflex in individuals is necessary to verify the effects of reducing the gag reflex [11].

In recent years, according to studies in various fields, such as medical care, nursing, and palliative care, aromatherapy has become a complementary and alternative therapy aimed at the prevention and treatment of diseases [12, 13]. Particularly, aromatherapy using essential oils (e.g., peppermint) has been reported to be effective in suppressing postoperative vomiting [14, 15]. In dentistry, aromatherapy has been reported to help patients feel relaxed during dental treatment and reduce anxiety [16–18]. Generally, aromatherapy is administered via a diffuser in the dental office or waiting room to help patients relax during treatment. However, to our knowledge, there are no previous reports on the effect of aromatherapy on the gag reflex during dental treatment. If aromatherapy can help suppress the gag reflex and allow successful completion of dental procedures, this would be beneficial for both the patient and the dentist. A well-designed and well-reported trial evaluating the effectiveness of aromatherapy in controlling the gag reflex is required.

This study aimed to determine whether aromatherapy reduces the gag reflex during dental procedures. Hence, we conducted this study to examine the anti-gag reflex effect of essential oils using objective and physiological evaluations of the gag reflex. We hypothesized that essential oils would be more effective than placebo in reducing the gag reflex.

## Methods

### Study design

This randomized, placebo-controlled, single-blind crossover trial was conducted in the Nippon Dental University, School of Life Dentistry at Tokyo, Japan. The study protocol was approved by the Ethics Committee of Nippon Dental University, School of Life Dentistry (NDU-T2016-13) and conforms to the guidelines of the Declaration of Helsinki. Before the study commenced, each participant was given comprehensive instructions on the study’s objectives and the procedures to be followed. Informed consent was obtained from all participants prior to their inclusion. This study was based on the CONSORT guidelines. The study was also registered in the University hospital Medical Information Network Clinical Trials Registry under the code UMIN000050616 (approved 17/03/2023).

### Sample size

Since there are no reports on the effect of aromatherapy on the suppression of the gag reflex during dental treatment, the sample size was determined by means of a pilot study. Using the G*power program (ver.3.1.9.2) [19], with an effect size of 0.5, and a paired t-test, we determined that this study required at least 17 participants (α = 0.05, β = 0.20).

### Participants

Twenty-four healthy Japanese adults (12 females, 12 males; average age 34.3 ± 9.5 years old) who consented to participate in the study were selected. The eligibility criteria for the participants were as follows: (1) adults aged 20–50 years, (2) history of previous dental treatment, and (3) no serious medical history. We excluded participants with (1) mental illness, physical disease, or oral disease; (2) dentures; (3) a history of eustachian tube obstruction, nasal obstruction, pneumothorax, or intestinal obstruction; and (4) medication prescriptions that may affect autonomic nerve activity. All participants were recruited from the surrounding areas through websites. They were given an honorarium (approximately 4,000 JPY) after completion of the study.

### Psychological evaluation

Psychological assessments were performed using four self-reported questionnaires. The Dental Fear Survey (DFS) [20] was used to assess dental fear levels. This questionnaire consists of 20 items that addressed anxiety-provoking situations during dental procedures. The items are rated from low (score 1) to high (score 5), with the total score ranging from 20 to 100 points. Higher scores indicate the presence of greater dental fear. We used the Level of Exposure-Dental Experiences Questionnaire (LOE-DEQ-16) [21] to assess the participants’ backgrounds regarding previous exposure to distressing dental events. The questionnaire consists of 16 items related to painful dental situations experienced in the past. For each of the 16 items, the participants were asked to indicate whether they had never (score 0) or ever (score 1) been exposed to an event. Each item of the LOE-DEQ was scored and summed to obtain an overall score, ranging from 0 to 16. Higher scores indicate past exposure to highly distressing dental event. The Dental Coping Strategy Questionnaire (DCSQ-15) was used to evaluate the participants’ coping strategies [22]. The participants were asked about the usage frequency of the 15 coping strategies in dental treatment situations. The items were scored on a 7-point Likert scale, where 1 indicates “never used” and 7 indicates “always used”, with a total score ranging from 15 to 105. Higher scores indicate more frequent use of coping strategies during dental treatment. The Gagging Assessment Scale (GAS) [23] was used to assess the degree of nausea during dental treatment. The GAS consists of four questions in the order of likelihood of inducing nausea and vomiting. The total GAS score ranges from 4 to 20. Higher scores are indicative of a higher tendency to vomit.

### Gag reflex evaluation

The gag reflex was objectively evaluated using a previously established method [11]. We used a standard disposable polyvinyl chloride saliva ejector (length, 143 mm; diameter, 6.5 mm; Premium Plus Japan Co., Osaka, Japan) with a stopper made of add-on silicone impression putty (Fusion II putty type; GC Co., Tokyo, Japan). The saliva ejector was directed from the maxillary central incisor to the pharynx and slowly inserted along the palate (10 mm/s) by the examiner. The participants pressed a cue button to inform the examiner when they reached the nausea threshold, after which the examiner removed the saliva ejector. To avoid measurement bias, another examiner measured the insertion distance of the saliva ejector using a Digital Vernier Caliper (Digital Vernier Caliper, 19,975; Shinwa Measurement Co., Niigata, Japan). This distance was used as the gag reflex value.

### Assignment of participants

**Fig. 1:**
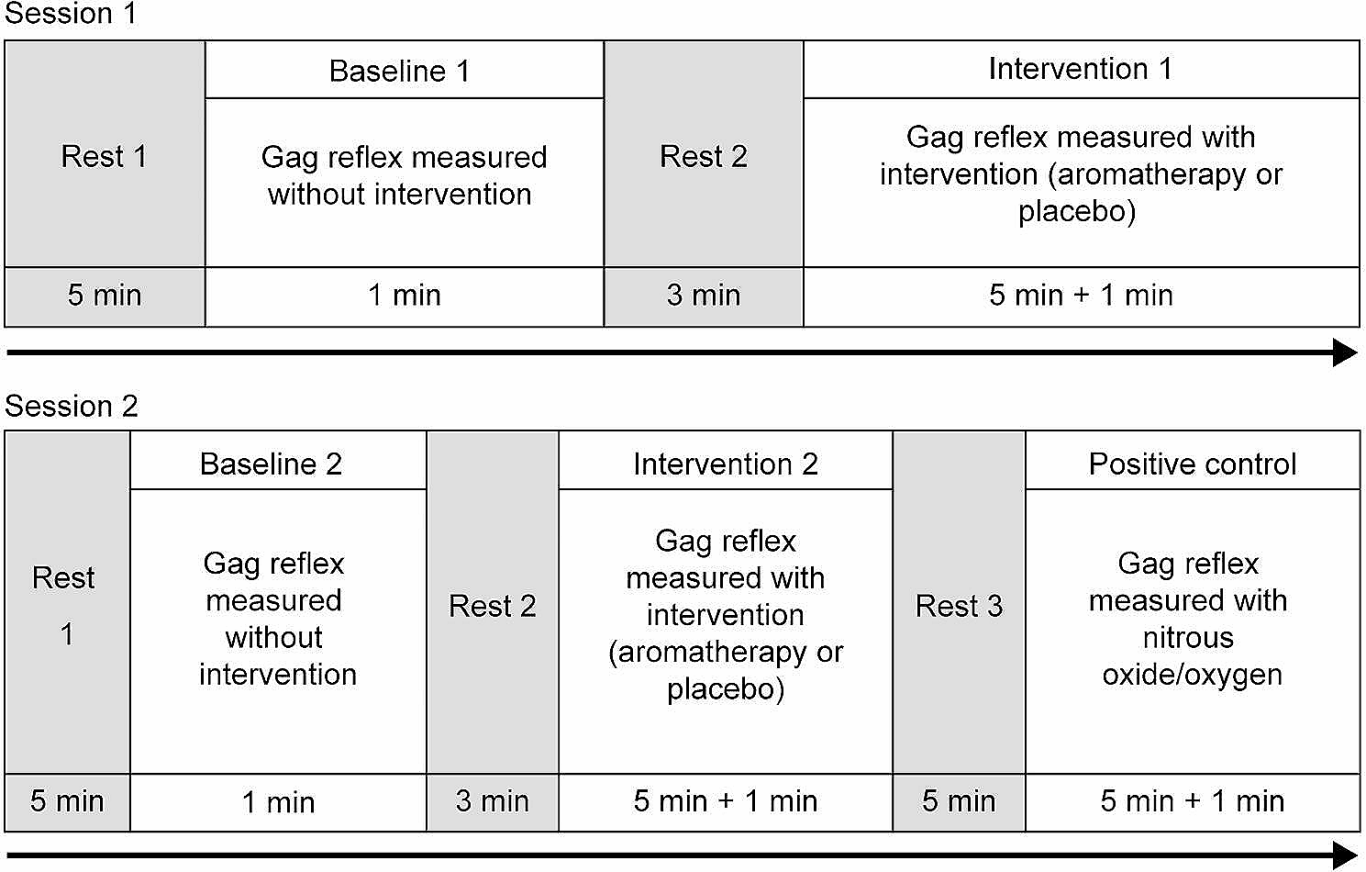
Time course of the study protocol

The protocol used in this study is illustrated in Fig. 1. The participants participated in two sessions (sessions 1 and 2) and received randomized interventions. The intervention was randomized into two sessions according to a stratified randomization assignment table created by a computerized number generator. First, participants were assigned a serial number in the order of their participation by sex. Then, the results of the stratified randomization assignment table corresponding to the serial number were determined. Participants assigned an odd number in the stratified randomization assignment table were to undergo aromatherapy using peppermint essential oil in session 1 and a placebo intervention using distilled water in session 2. Those assigned an even number received placebo in session 1 and aromatherapy in session 2. This information was maintained by a principal investigator and was not shared with participants or examiners.

The interval between sessions 1 and 2 was 2 weeks, based on the results of our preliminary study on daily fluctuations in gag reflex evaluation. Gag reflex measurements of six subjects showed no significant differences between baseline, 1-week, and 2-week measurements (repeated-measures analysis of variance [ANOVA], *P* = 0.45), with an intraclass correlation coefficient of 0.975. These subjects in the preliminary study were not included in the present study.

### Time schedule

All studies were conducted in a quiet room under constant environmental conditions (room temperature, 21–27℃; humidity, 25–75%; illuminance, 300–380 lx), with the participants seated in a reclining chair. Participants were restricted from eating or drinking within 2 h prior to the measurement in order not to affect the measurement of the gag reflex. Measurement times for the same participant were adjusted to the same time between 9 am and 5 pm across sessions.

In session 1, the gag reflex value was measured before the intervention, and this measurement was used as the baseline measurement. Next, as per the materials used in previous studies [17, 24], a filter paper blotted with 150 µL of peppermint essential oil (Mentha × piperita; Mont St-Michel, France) or distilled water (Japanese Pharmacopoeia, Water for Injection, Otsuka Distilled Water: Otsuka Pharmaceutical Co., Tokyo, Japan) was fixed to a transparent mask (length = 107 mm, width = 175 mm; Asahi Sogyo Co., Osaka, Japan) and adjusted to come to the participant’s nose tip. The participants were asked to wear the mask in a manner that it did not touch their skin, and the filter paper was placed on the tip of their nose for 5 min [17, 24]. Subsequently, the gag reflex value was measured and used as the post-intervention value. In session 2, the baseline gag reflex values and post-intervention gag reflex values were measured in a similar manner. Furthermore, in session 2, after the post-intervention measurement, the participants were administered 30% nitrous oxide/70% oxygen by inhalation for 5 min as a positive control, followed by measurement of the gag reflex value.

Before starting the baseline measurements for each session, the participants were asked to rest for 5 min; there was a 3-min rest period between the baseline and intervention to reduce the carryover effect of the gag reflex assessment. In addition, after the intervention in session 2, there was a 5-min rest period during which the participants inhaled 100% oxygen through a nasal mask for 3 min to wash out the effects of the intervention. Afterwards, a positive control measurement was performed.

In this study, the participants were not provided information about the intervention being used. However, differences in scent between peppermint essential oil and distilled water may make it difficult to ensure blinding for participants. Meanwhile, the examiner who measured the gag reflex value wore a mask containing activated charcoal to conceal the scent and ensure blinding. Therefore, this study was conducted as a single-blind study.

### Subjective ratings of the scents

After each session, the participants subjectively rated the peppermint essential oil and distilled water. The participants were asked to subjectively evaluate the scents they were exposed to in the previous intervention on a visual analog scale (VAS; 0–100 mm) for the following items: valence (0 [very unpleasant], 100 [very pleasant]), activation (0 [very calm], 100 [very aroused]), happiness, disgust, fear, and pleasure (0 [not at all], 100 [very strong]) [25–27].

### Physiological evaluation

Pulse rate and galvanic skin response (GSR) were recorded for each session. The pulse rate was recorded using a pulse transducer attached to the medial phalanx of the thumb of the non-dominant hand. LabChart 8 software (ADInstruments, Sydney, Australia) was used to identify the peak slopes and study pulse discrimination. The signals were sampled at 1 kHz and filtered using a 50-Hz low-pass filter. The GSR was recorded using specific GSR sensors with a signal amplifier (GSR Amp FE116; ADInstruments) and a high-performance data acquisition system (PowerLab 8/35, PL3508; ADInstruments). The sensors were attached to the medial phalanges of the index and middle fingers. Signals were sampled at 1 kHz and recorded using LabChart 8 software.

For each session, the average pulse rate values for 30 s before and 30 s after insertion of the saliva ejector were calculated and compared. The rate of change in the GSR for 30 s before and 10 s after insertion of the saliva ejector was calculated and compared between the baseline and that at each intervention [28]. To remove negative scores from the raw GSR data, they were converted to T-scores with a mean of 50 and standard deviation of 10.

### Statistical analysis

The Shapiro–Wilk test was used to confirm the normality of the dataset. Student’s t-tests were used to compare the psychological evaluations between sexes. Repeated-measures ANOVA was used to compare the pre-intervention gag reflex values among interventions. A paired t-test was used to compare the gag reflex, pulse rate, and GSR change rates before and after each intervention with the baseline values. Wilcoxon signed-rank test was used to compare the subjective ratings for peppermint essential oil and distilled water. Repeated-measures ANOVA was used to compare the rate of increase in gag reflex values between the interventions, and multiple comparisons were performed using Tukey’s honest significant difference test. A *p*-value of less than 5% was considered statistically significant. All analyses were performed using SPSS Statistics for Windows (version 21.0; IBM, Armonk, NY).

## Results

### Characteristics of participants

Table 1 shows the ages of the participants and the means and standard deviations of their psychological evaluations. Examination of the differences between sexes revealed significant differences in the LOE-DEQ-16 and GAS scores, but no significant differences were found in other items.

**Table 1:** Characteristics of participants (mean ± SD)

	Females (*n* = 12)	Males (*n* = 12)	*P* value*	Total (*n* = 24)
Age (years)	33.5 ± 10.4	35.2 ± 8.8	0.68	34.3 ± 9.5
DFS score	48.1 ± 18.8	35.3 ± 13.7	0.07	41.7 ± 17.4
LOE-DEQ-16 score	3.1 ± 1.9	1.4 ± 1.8	0.041	2.3 ± 2.0
DCSQ-15 score	47.6 ± 13.6	49.9 ± 18.6	0.73	48.8 ± 16.0
GAS score	6.0 ± 1.7	4.1 ± 0.3	0.002	5.0 ± 1.5

* Student’s t-test, SD: standard deviation, DFS: Dental Fear Survey, LOE-DEQ: Level of Exposure-Dental Experiences Questionnaire, DCSQ: Dental Coping Strategy Questionnaire, GAS: Gagging Assessment Scale

### Comparison of gag reflex values

The mean pre-intervention gag reflex values were 83.6 ± 11.8 mm, 83.0 ± 13.4 mm, and 83.8 ± 12.6 mm for placebo, aromatherapy, and positive control, respectively, with no significant difference (repeated-measures ANOVA, *P* = 0.67). The gag reflex values significantly increased after the intervention compared to the baseline values for the placebo intervention, aromatherapy, and positive control (paired t-test, *P* = 0.01, *P* < 0.001, and *P* < 0.001, respectively) (Table 2).

**Table 2:** Comparison of gag reflex values before and after each intervention (mean ± SD)

Intervention	Baseline (mm)	After intervention (mm)	*P* value*
Placebo	83.6 ± 11.8	86.4 ± 13.5	0.01
Aromatherapy	83.0 ± 13.4	89.8 ± 14.6	<0.001
Positive control	83.8 ± 12.6	89.7 ± 13.2	<0.001

* Paired t-test; SD: standard deviation

### Subjective ratings of the scents

Table 3 shows the subjective ratings for peppermint essential oil and distilled water treatments. The comparison of the subjective ratings revealed significant differences in valence, activation, disgust, and pleasure (Wilcoxon signed-rank tests; *P* = 0.004, 0.01, 0.03, and 0.04, respectively).

**Table 3:** Subjective ratings of scents (mean ± SD)

	Peppermint essential oil (mm)	Distilled water (mm)	*P* value*
Valence	67.3 ± 15.4	54.8 ± 9.3	0.004
Activation	37.3 ± 24.6	21.9 ± 17.1	0.01
Happiness	48.0 ± 26.1	34.9 ± 26.7	0.18
Disgust	16.1 ± 22.3	7.2 ± 13.6	0.03
Fear	5.5 ± 10.9	5.4 ± 11.3	0.88
Pleasure	61.7 ± 24.7	47.3 ± 27.0	0.04

*Wilcoxon signed-rank test; VAS: visual analog scale; SD: standard deviation; VAS: 0–100 mm

### Comparison of rate of increase in gag reflex value for each intervention

A significant difference was observed between the rates of increase in the gag reflex values before and after the intervention for all three interventions (repeated-measures ANOVA, *P* = 0.003) (Table 4). Multiple comparisons showed no significant difference between the aromatherapy and the positive control (*P* = 0.83), but there was a significant difference between the aromatherapy and placebo intervention, and between the placebo intervention and positive control (*P* = 0.004 and 0.02, respectively).

**Table 4:** Comparison of rate of increase in gag reflex value for each intervention (mean ± SD)

Intervention	Increase rate of gag reflex values from baseline (%)	*P* value*
Placebo	3.3 ± 6.5^a^	
Aromatherapy	8.3 ± 7.6^b^	0.003
Positive control	7.4 ± 8.0^b^	

* Repeated measures analysis of variance; SD: standard deviation

Lowercase letters correspond to differences among the three groups within the column using post-hoc comparisons (aromatherapy vs. positive control, *P* = 0.83; aromatherapy vs. placebo, *P* = 0.004; placebo vs. positive control, *P* = 0.02)

### Physiological evaluation

On comparing the mean pulse rates 30 s before and after insertion of the saliva ejector, a significant increase was observed after insertion at the baseline and at the placebo intervention (paired t-test, *P* = 0.002 and 0.04, respectively); however, no significant changes were observed in aromatherapy or positive control intervention (Table 5).

**Table 5:** Comparison of pulse rate before and after gag reflex measurement in each intervention (mean ± SD)

Intervention	Before gag reflex measurement (bpm)	After gag reflex measurement (bpm)	*P* value*
Baseline	70.1 ± 10.7	72.7 ± 12.5	0.002
Placebo	68.8 ± 11.4	73.5 ± 15.3	0.04
Aromatherapy	72.5 ± 9.8	72.0 ± 7.2	0.18
Positive control	63.1 ± 8.3	65.8 ± 10.2	0.07

* Paired t-test; SD: standard deviation; bpm: beats per minute

On comparing the rate of change in the GSR 30 s before and 10 s after the insertion of the saliva ejector between the baseline and each intervention, no significant difference was observed between the baseline and placebo intervention and positive control. Aromatherapy resulted in significantly fewer changes in the GSR than the baseline GSR changes (paired t-test, *P* = 0.008) (Table 6).

**Table 6:** Comparison of rate of change in GSR before and after each intervention (mean ± SD)

Intervention	Baseline (%)	After intervention (%)	*P* value*
Placebo	131.1 ± 21.1	120.8 ± 23.0	0.11
Aromatherapy	137.2 ± 25.1	117.7 ± 23.7	0.008
Positive control	127.4 ± 22.1	123.2 ± 26.2	0.55

* Paired t-test, GSR: galvanic skin response, SD: standard deviation

## Discussion

We evaluated the suppression of the gag reflex when smelling essential oils using objective and physiological indices of the gag reflex. Aromatherapy using essential oils was found to have a higher anti-gag reflex effect than the placebo, supporting our hypothesis.

The participants were healthy Japanese adults who had undergone dental treatment. Fear of dental treatment is generally higher in women than in men [29]. In this study, the DFS tended to be higher among females (mean 48.1) than among males (mean 35.3); however, no significant difference was observed. The average DFS of the Japanese population is estimated to be 37.4 [30]. Thus, some female participants may have had stronger dental fear. On the LOE-DEQ-16, females (mean 3.1) showed significantly higher scores than males (mean 1.4), but the difference was small. As unpleasant experiences from prior dental treatments might indirectly exacerbate a strong gag reflex [31], each participant’s history of dental treatment was evaluated by the LOE-DEQ-16. The mean LOE-DEQ-16 was 6.9 in general dental patients in a previous study [21]. However, the present study demonstrated a lower mean LOE-DEQ-16 score of 2.3 for all participants compared to that of the previous study. This suggests that the participants in the current study did not experience much pain during past dental treatment. No sex differences were observed in the DCSQ-15 in the present study. In a previous study, the DCSQ-15 was administered to 94 fearful dental patients undergoing routine dental care, with a mean value of 55.5 [22]. As per the DCSQ-15 score in the present study (mean, 48.8), the participants appeared to use few coping strategies for dental treatment. Regarding the GAS, females (mean 6.0) tended to experience significantly higher levels of nausea than males (mean 4.1). However, the degree of gagging was not as severe as the average value of 6.4 in a previous study [23]. Therefore, the participants in the current study had a strong fear of dental treatment but did not have a severe gag reflex.

This study used a crossover design to ensure the inclusion of an appropriate number of participants. Participants attended two sessions separated by a maximum of 2 weeks. In this study, positive control intervention was administered at the end of session 2, and no new session was allocated for it. Therefore, it is unclear if there was a carryover effect from the immediately preceding aromatherapy/placebo intervention. However, in our preliminary study, we compared gag reflex values before aromatherapy intervention and after 5 min of aromatherapy followed by 3 min of oxygen inhalation and observed no significant differences (paired t-test, *P* = 0.68). Thus, a positive control was allocated at the end of session 2 after a 3-min post-intervention oxygenation to wash out any carryover effects.

There were no significant differences in the baseline gag reflex values in each session, suggesting that each session was measured under similar conditions. Unexpectedly, the gag reflex values significantly increased after the placebo, aromatherapy, and positive control interventions compared to the baseline values, indicating that all interventions were effective in suppressing the gag reflex. The effect of a scent may be influenced by whether its rating is positive or negative. In this study, subjective ratings of scents were performed by applying the affective reaction with reference to previous studies [25–27]. We used valence and activation as participants’ affective reactions to scents. Additionally, happiness and pleasure were used to evaluate positivity toward the scents, and disgust and fear were used to evaluate negativity. On comparing the participants’ ratings of peppermint essential oil and distilled water, peppermint was rated with significantly higher values for valence, activation, disgust, and pleasure than distilled water, suggesting that the participants perceived the scent of peppermint to be stronger than that of distilled water. However, no significant difference was observed in the subjective happiness ratings between them. The mean happiness and pleasure values for distilled water were 34.9 and 47.3, respectively. These two ratings were considerably high even for odorless water. In a randomized controlled trial, Diep et al. [10] compared the gag reflex suppressive effects of acupuncture and transcutaneous electrical acupoint stimulation with a sham placebo. The authors observed no significant differences in the suppressive effects of the three treatments. The reason behind this is the marked placebo effect associated with the expectation of improvement in true reductions in the gag reflex through subjective and central mechanisms. Gag reflex is associated with anxiety and stress [4]. The distilled water intervention in the present study may have led to increased gag reflex values because of the placebo effect of participating in a study on the gag reflex.

On comparing the rate of increase in the gag reflex values for each intervention, a significant difference was observed between the aromatherapy and placebo conditions and between the positive control and placebo conditions. However, no significant difference was found between the aromatherapy and positive control conditions. This suggests that placebo intervention was not as effective as aromatherapy in suppressing the gag reflex, whereas peppermint essential oil was as effective as nitrous oxide inhalation in suppressing the gag reflex.

The main components of peppermint essential oil are menthol, menthone, and menthyl acetate [32]. Aromatherapy with peppermint essential oil is thought to have the effect of suppressing the gag reflex due to the antispasmodic action of menthol contained in the oil, resulting in the relaxation of the esophageal sphincter muscle [33]. Additionally, the serotonin 5-HT3 receptors in the brain chemoreceptor trigger zone and gastrointestinal tract transmit the stimulation (sensory input) to the vomiting center via serotonin. Peppermint essential oil has an antagonistic effect on the 5-HT3 receptor channel that inhibits the stimulation of the vomiting center, thereby suppressing the gag reflex [34].

Oral stimulation may be accompanied by perspiration and increased heart rate owing to the proximity of the vasomotor and cardiac centers to each other [1]. The results of the physiological assessment indicated the stability of autonomic responses to aromatherapy. The baseline and placebo interventions significantly increased the pulse rate when the gag reflex values were measured, whereas aromatherapy and positive control treatment did not. Jafarzadeh et al. [35] reported a significant decrease in pulse rate with aromatherapy intervention with orange essential oil compared to control treatment during pediatric dental treatment. This finding is inconsistent with the results of the present study, where no significant reduction was observed with the intervention. In addition, the rate of change in the GSR from before to after the measurement of the gag reflex value did not show a significant difference between the placebo intervention and positive control conditions compared to the baseline GSR changes, but it significantly decreased with aromatherapy. However, in all interventions, the GSR increased after measuring the gag reflex value. Thus, it is difficult to conclude whether a sedative effect predominantly acting on the parasympathetic nerve was observed. Ghaderi et al. [18] reported that during the dental procedures, compared with the control condition, aromatherapy with lavender essential oil led to reduced anxiety and pain in pediatric patients. The authors stated that the scent of lavender reduced anxiety and increased sedation by stimulating the parasympathetic nervous system. Although the peppermint essential oil used in the present study did not have a sedative effect, different effects can be expected depending on the type of essential oil used for aromatherapy in dental practice.

This study had some limitations. First, the aromatherapy method used in this study was different from that used in the original technique. In our study, a filer paper with undiluted aromatic essential oil was placed on the tip of the nose for 5 min, and the scent from it effectively suppressed the gag reflex. However, it is unclear whether aromatherapy using a diffuser has the same effect on suppressing the gag reflex in patients. In the future, it will be necessary to develop safer and more effective methods for inhaling scents. Second, the participants in this study exhibited relatively few gag reflexes during dental treatment; therefore, the effect of our intervention in patients with a strong gag reflex remains unknown. Future studies with a large number of participants with strong gag reflexes are needed to verify the suppressive effects of aromatherapy.

## Conclusions

This study examined the effect of aromatherapy on gag reflex inhibition during dental treatment. These results suggest that aromatherapy with peppermint essential oil has an inhibitory effect on the gag reflex during dental treatment, comparable to that of inhalation of nitrous oxide. This may serve as a non-invasive method for patients with a gag reflex. However, further clinical trials are required to confirm the efficacy of the diffuser-based method and its effectiveness in patients with strong gag reflexes before it can be used in dental practice.

## Data Availability

The datasets generated/analyzed during the current study are available from the corresponding author on reasonable request.

## Abbreviations

DFS: Dental Fear Survey
LOE-DEQ: Level of Exposure-Dental Experiences Questionnaire
DCSQ: Dental Coping Strategy Questionnaire
GAS: Gagging Assessment Scale
GSR: Galvanic Skin Response
ANOVA: Analysis of variance
